# Identification of functional mutations associated with environmental variance of litter size in rabbits

**DOI:** 10.1186/s12711-020-00542-w

**Published:** 2020-05-06

**Authors:** Cristina Casto-Rebollo, María José Argente, María Luz García, Romi Pena, Noelia Ibáñez-Escriche

**Affiliations:** 1 Institute for Animal Science and Technology, Universitat Politècnica de València, Valencia, Spain; 2 Departamento de Tecnología Agroalimentaria, Universidad Miguel Hernández de Elche, Orihuela, Spain; 3 Departament de Ciència Animal, Universitat de Lleida-AGROTECNIO Center, Lleida, Catalonia Spain

## Abstract

**Background:**

Environmental variance (V_E_) is partly under genetic control and has recently been proposed as a measure of resilience. Unravelling the genetic background of the V_E_ of complex traits could help to improve resilience of livestock and stabilize their production across farming systems. The objective of this study was to identify genes and functional mutations associated with variation in V_E_ of litter size (LS) in rabbits. To achieve this, we combined the results of a genome-wide association study (GWAS) and a whole-genome sequencing (WGS) analysis using data from two divergently selected rabbit lines for high and low V_E_ of LS. These lines differ in terms of biomarkers of immune response and mortality. Moreover, rabbits with a lower V_E_ of LS were found to be more resilient to infections than animals with a higher V_E_ of LS.

**Results:**

By using two GWAS approaches (single-marker regression and Bayesian multiple-marker regression), we identified four genomic regions associated with V_E_ of LS, on chromosomes 3, 7, 10, and 14. We detected 38 genes in the associated genomic regions and, using WGS, we identified 129 variants in the splicing, UTR, and coding (missense and frameshift effects) regions of 16 of these 38 genes. These genes were related to the immune system, the development of sensory structures, and stress responses. All of these variants (except one) segregated in one of the rabbit lines and were absent (n = 91) or fixed in the other one (n = 37). The fixed variants were in the *HDAC9*, *ITGB8*, *MIS18A*, *ENSOCUG00000021276* and *URB1* genes. We also identified a 1-bp deletion in the 3′UTR region of the *HUNK* gene that was fixed in the low V_E_ line and absent in the high V_E_ line.

**Conclusions:**

This is the first study that combines GWAS and WGS analyses to study the genetic basis of V_E_. The new candidate genes and functional mutations identified in this study suggest that the V_E_ of LS is under the control of functions related to the immune system, stress response, and the nervous system. These findings could also explain differences in resilience between rabbits with homogeneous and heterogeneous V_E_ of litter size.

## Background

Understanding the effect of the environment on the phenotype of farm animals is important to improve responses to genetic selection. The environment can affect both the mean of a trait and its variance (environmental variance or ${\text{V}}_{\text{E}}$). Many studies in various species have provided statistical evidence that ${\text{V}}_{\text{E}}$ is partly under genetic control: pigs [1], mice [2], chickens [3], snails (*Helix aspersa*) [4] and cattle [5], among others. For instance, the ${\text{V}}_{\text{E}}$ can differ between genotypes under the same environment [6]. Successful divergent selection experiments for ${\text{V}}_{\text{E}}$ support these findings in both livestock and model animals [7–9].

Recently, ${\text{V}}_{\text{E}}$ was proposed as a measure of resilience [10], which is the ability of an animal to maintain or quickly recover their performance in spite of environmental perturbations [11, 12]. Previous genome-wide association studies (GWAS) for ${\text{V}}_{\text{E}}$ have identified relevant contributions from candidate genes that are related to important phases of the inflammatory response, such as *Hsp90* [13, 14], *p22*-*PHOX*, *GNG11,* and *GNGT1* [15], which are triggered by tissue damage and the entry of pathogens [16]. In humans, the *FTO* gene, which affects the variability of body mass index [17], was also found to be associated with sensitivity to infections [18]. All these results support the role of the immune system in the detection and response to environmental perturbations such as pathogen infections [12].

Unravelling the genetic background of the ${\text{V}}_{\text{E}}$ of complex traits could help to improve resilience of livestock and stabilize their production across farming systems [19]. The aim of this study was to identify genes and functional mutations associated with variation in the ${\text{V}}_{\text{E}}$ of litter size (LS) in rabbits. We performed a GWAS and a whole-genome sequencing (WGS) analysis using data from two rabbit lines that have been divergently selected for high and low ${\text{V}}_{\text{E}}$ of LS [9]. These lines show a remarkable difference in ${\text{V}}_{\text{E}}$ of LS (4.5% of the mean of the base population), as well as differences in mortality, in biomarkers of immune response (plasma cortisol, leukocytes and acute-phase protein levels), and in plasma concentrations of cholesterol and triglycerides [20]. Moreover, the line with a low ${\text{V}}_{\text{E}}$ of LS was found to cope better with environmental stressors such as infections than the line with a high ${\text{V}}_{\text{E}}$ of LS, which suggests that the homogeneous line is more resilient.

## Methods

### Animals

The rabbits used in this study belong to a divergent selection experiment for high and low ${\text{V}}_{\text{E}}$ of LS over 12 generations at the University Miguel Hernandez of Elche, Spain. Each divergent line had approximately 125 female and 25 male parents per generation (for more details see Blasco et al. [9]). The total number of litters over these generations was equal to 13,788 for 3070 does: 6094 from the line with a low ${\text{V}}_{\text{E}}$ of LS, 6682 from the line with a high ${\text{V}}_{\text{E}}$ of LS, and 1012 from the base population. In total, 1658 records of litter size from generations 11 and 12, and genotypes for 384 does were used for the GWAS: 96 from the base population (404 parities), 149 from the line with a high ${\text{V}}_{\text{E}}$ of LS (649 parities), and 139 from the line with a low ${\text{V}}_{\text{E}}$ of LS (605 parities). The average litter size (total number born; TNB includes live born plus stillborn) for the base population and the lines with a low and high ${\text{V}}_{\text{E}}$ of LS was 8.72 (± 3.05), 7.71 (± 2.38) and 6.51 (± 3.06), respectively.

### Phenotype

In this study, we investigated genomic regions that were associated with the ${\text{V}}_{\text{E}}$ of LS, which was the selection criterion in the divergent selection experiment [9]. The ${\text{V}}_{\text{E}}$ of LS was calculated as the within-doe variance of TNB, after correction of TNB by year-season (47 levels) and parity-lactation status (3 levels) to avoid the effect of systematic effects on ${\text{V}}_{\text{E}}$. The mean estimate of residuals for a doe across parities was used to calculate the ${\text{V}}_{\text{E}}$ of LS for a doe, using the minimum quadratic risk estimator:

1 $${\text{V}}_{\text{E}} = \frac{1}{n + 1}\mathop \sum \limits_{1}^{n} \left( {x_{i} - \bar{x}} \right)^{2} ,$$

where $x_{i}$ is the pre-corrected TNB at parity $i$ of a doe and $n$ is the total number of parities of the doe (ranging from 2 to 12). ${\text{V}}_{\text{E}}$ was calculated by assuming that the additive genetic and permanent effects are approximately the same for each parity of a doe [21]. The average of the ${\text{V}}_{\text{E}}$ of LS was 4.24 (± 3.41), 2.27 (± 1.97) and 3.84 (± 3.69) for the base population and for the low and high lines, respectively.

### Genotypes

Genomic DNA was isolated from blood sampled from does using standard procedures. Genotyping was performed with the 200 K Affymetrix Axiom OrcunSNP array (ThermoFisher Scientific). Quality control of genotypes was performed using the platform Axiom Analysis Suite 3.1 of ThermoFisher Scientific and the PLINK v1.9 software [22]. Animals with a call rate lower than 97% and SNPs with a minor allele frequency lower than 0.05, with missing genotypes higher than 0.05, or with unknown positions on the rabbit reference genome (OryCun v2.0.96) were removed. After quality control, 367 animals (1589 parities) and 96,329 SNPs remained in the dataset. The missing genotypes were imputed with the Beagle v.4.1 software [23]. Finally, we identified outliers and checked the population structure by applying a principal component analysis (PCA) based on the genotypes (see Additional file 1).

### GWAS

Two approaches were used for GWAS: single-marker regression (SMR) and Bayesian multiple-marker regression (BMMR). SMR was performed using the linear mixed model method, which is available in the GCTA v1.91.4 beta software [24]. To correct for population stratification, GCTA considers the genomic relationship matrix built, but without SNPs on the chromosome of the tested SNP [25]. The SNPs that were associated with ${\text{V}}_{\text{E}}$ of LS were identified at a conservative p-value threshold of 0.0001 [26]. Using the same method, we also tested the effect of ignoring differences in number of parities between does. In order to do that, we performed the SMR using a weighted linear mixed model method implemented in the R software. Instead of a genomic relationship matrix, the model included the first five principal components of the genomic relationship matrix to correct for population stratification. The ${\text{V}}_{\text{E}}$ of LS was weighted according to Blasco et al. [9]:

2 $$w_{i} = \frac{{\left( {n_{i} + 1} \right)^{2} }}{{2*\left( {n_{i} - 1} \right)}},$$

where $n_{i}$ is the total number of parities for doe $i$.

BMMR was performed using a Bayes B model that is implemented in the GenSel software [27]. This model assumed that, in a given iteration of the Monte Carlo Markov chain, many SNPs have no effect and variance, with a prior probability of π = 0.999, and approximately 100 SNPs have an effect and a variance on the ${\text{V}}_{\text{E}}$ of LS. The analysis was done using a chain length of 550,000, with a lag of 100 and a burn-in of 150,000. The means of the priors of the genotypic and environmental variances were equal to 5.1 and 4.3, respectively. A Bayes factor was calculated to determine statistical significance of the SNP associations, as:

$$BF = \frac{{\hat{p}_{i} /\left( {1 - \hat{p}_{i} } \right)}}{{\left( {1 - \pi } \right)/\pi }},$$

where $\pi$ is the prior probability and $\hat{p}_{i}$ is the posterior probability of a SNP in locus $i$ having an effect. A threshold for $BF$ higher than 10 was used to identify SNPs that are associated with ${\text{V}}_{\text{E}}$ of LS [28]. The contribution of each of the 2125 non-overlapping 1-Mb genomic windows to the genetic variance was computed as the posterior mean of the percentage of the genomic variance explained by all markers across the genome (total genomic variance).

#### Additional evidence for associated SNPs

The SNPs identified to be associated with ${\text{V}}_{\text{E}}$ using both the SMR and BMMR approaches were further tested using a permutation test and a GWAS within each population (base, with a high and with a low ${\text{V}}_{\text{E}}$ of LS) to determine whether they were spurious associations due to drift. Only SNPs that passed these additional tests were considered as displaying a true association with ${\text{V}}_{\text{E}}$ of LS.

The permutation test was performed using the PLINK v1. 9 software [22]. In total, 100,000 random permutations were performed to remove the true association between ${\text{V}}_{\text{E}}$ of LS and the genotype. Each permutated dataset was analysed using a linear-mixed model and the p-value of each SNP was calculated. The resulting distribution of p-values was used to calculate an empirical p-value (EMP1) for each SNP in the original data based on the number of times that the p-value of that SNP was declared to have a significant association with ${\text{V}}_{\text{E}}$ of LS under the null hypothesis of no association in the permutated data. The minimum EMP1 that could be registered was 1/N, where N is the number of permutation tests. Thus, only SNPs with an EMP1 close to 0.00001 were considered to be associated with ${\text{V}}_{\text{E}}$ of LS.

Within-population GWAS was performed for each population using the SMR approach [24]. The same reference alleles were established in the two lines and in the base population to estimate allele substitution effects. Confidence intervals (CI) of the SNP effects within a population were estimated as the allele substitution effect estimate ± 2SE. Overlapping CI for a SNP between lines was declared to signify no evidence of differences in allele effects on the phenotype across populations.

#### Identification of associated genomic regions

The significant SNPs were used to perform a linkage disequilibrium (LD) study using the PLINK v1.9 software [22]. For this purpose, SNPs within 0.5 Mb of a significant SNP were grouped into blocks, which were then expanded to genomic regions ± 1 Mb for the LD study. Genomic regions associated with the trait were considered to be blocks of SNPs with r^2^ higher than 0.7 between each other. We established this strong threshold following Vanliere et al. [29], who determined that two SNPs were dependent when r^2^ was equal or higher than 0.43.

### Whole-genome sequencing

To identify which variants were present in one line but not in the other, due to the selection process, a pool of DNA from the breeding males in the 10th generation was created for each line (27 animals per line) and sequenced with Illumina Technology at an average depth of 27×. These males were all fathers of animals from the 11th generation, which were used in the GWAS.

Pre-processing of the WGS data was performed following Elston [30]. The BWA algorithm [31] was used to index the OryCun v2.0.96 reference genome from the raw data. Illumina adapters and low-quality read ends were removed using Trimmomatic v0.39 [32]. The BWA-MEM algorithm was used to align reads to OryCun v2.0.96. The sorted BAM files were obtained by SAMtools [33]. Duplicates were marked using Picard MarkDuplicates [34].

Variant calling was performed using the GATK Best Practices pipeline [35] by applying GATK to the BAM files using HaplotypeCaller and GenotypeGVCF to obtain the raw VCF files for the high and low ${\text{V}}_{\text{E}}$ of LS lines. Variants were filtered using SelectVariant from GATK. Single nucleotide variants (SNVs) that were filtered out were labelled using VariantFiltration with the following filter expressions: QD < 2, FS > 60, MQ < 40, MQRankSum < −12.5, ReadPosRankSum < −8. INDELs were filtered out according to QD < 2, FS > 200, and ReadPosRankSum < −20. Finally, variants were annotated using the snpEFF software [36].

### Identification of candidate genes and functional mutations

The Ensembl release 97 database [37] was used to investigate candidate genes in the genomic regions associated with ${\text{V}}_{\text{E}}$ of LS, using OryCun 2.0.97 as the reference genome. SNVs and INDELs that were present in the genomic regions associated with ${\text{V}}_{\text{E}}$ of LS were also detected. Variants that segregated differently between the two lines and that had a greater impact on gene function were proposed as functional mutations for ${\text{V}}_{\text{E}}$ of LS. We considered that a variant had a greater impact if it affected: (a) the amino acid sequence of the protein (missense or frameshift mutations), (b) the UTR regions of the mRNAs, or (c) the splicing pattern of the transcripts. Genes that contained such possible functional mutations were identified as candidate genes for ${\text{V}}_{\text{E}}$ of LS were. The biological functions and the gene ontology of the candidate genes were reviewed in GeneCards [38].

## Results

### Genomic regions associated with ${\mathbf{V}}_{{\mathbf{E}}}$ of LS

**Fig. 1:**
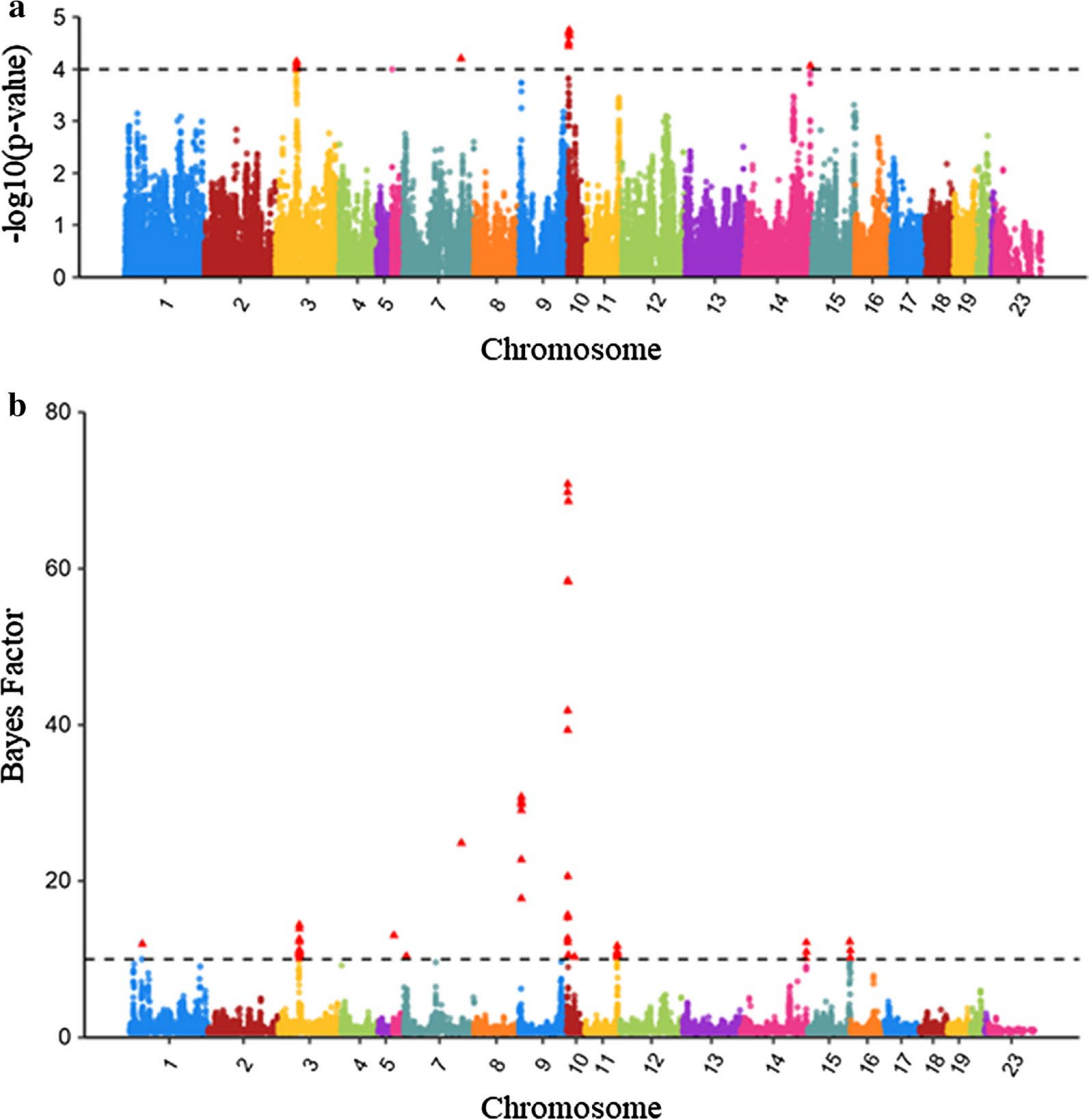
Manhattan plots for genome-wide association analyses for environmental variance of litter size. **a** −log_10_(p-value) for association of SNPs using the single-marker regression approach. **b** Bayes factor (BF) for association of SNPs using the Bayesian multiple-marker regression approach. The dashed lines represent significance thresholds a p-value of 0.0001 (**a**) and $BF$ of 10 (**b**). The red triangles highlight the SNPs that pass the threshold

GWAS identified SNPs associated with ${\text{V}}_{\text{E}}$ of LS using two approaches, SMR and BMMR. SMR identified 12 SNPs with a p-value less than 0.0001 on *Oryctolagus cuniculus* chromosomes (OCU) 3, 7, 10, and 14 (Fig. 1a). The same results were obtained with the weighted SMR analysis of ${\text{V}}_{\text{E}}$ to take differences in number of parities between does into account (data not shown). With BMMR, we identified 60 SNPs on several chromosomes that had a Bayes factor ($BF$) higher than 10 (Fig. 1b), including all the SNPs that were identified by SMR (Table 1). These latter SNPs were in genomic windows on OCU3 (50–52 Mb), OCU7 (141–142 Mb), OCU10 (4–5.7 Mb), and OCU14 (163–164 Mb), which explained 4.0, 0.2, 3.2 and 0.5% of the total genomic variance, respectively. The three most significant SNPs on OCU9 were also considered because they reached a p-value close to the threshold of 0.0001 (0.00018) and a $BF$ greater than 10 (Fig. 1). The genomic window that contained these SNPs on OCU9 (4–6 Mb) explained 0.9% of the total genomic variance. In summary, 15 SNPs were identified to be associated with ${\text{V}}_{\text{E}}$ of LS by both methods.

**Table 1:** Genomic regions associated with environmental variance of litter size in rabbits

OCU^a^	Position (Mb)	Significant SNPs	p-value	$\varvec{BF}$^b^	Genes located in the region
3	50.4–52.8	Affx-151987366	7.02e−5	13.88	*SPDL1*, *DOCK2*^c^, *INSYN2B*^c^, *FOXI1*^c^, *LCP2*, *KCNMB1*, *ENSOCUG00000020826*, *KCNIP1*, *GABRP*, *RANBP17*, *TLX3*, *FGF18*^*c*^*, ENSOCUG00000022678*, *ENSOCUG00000011117*^c^, *ENSOCUG00000018666*
		Affx-151799106	7.66e−5	14.39	
		Affx-151959457	8.79e−5	12.54	
7	141.2	Affx-151820818	6.26e−5	24.88	*AOX1*
10	3.9–5.7	Affx-151981327	1.76e−5	58.37	*HDAC9*^c^, *FERD3L*^c^, *TWISTNB*, *TMEM196*^c^, *ENSOCUG00000019989*, *ENSOCUG00000018779*, *MACC1, ITGB8*^c^
		Affx-151890261	1.97e−5	58.48	
		Affx-151932936	2.29e−5	69.79	
		Affx-151906185	2.29e−5	70.82	
		Affx-151891719	2.29e−5	68.65	
14	162–163.2	Affx-151919621^d^	8.65e−5	10.01	*HUNK*^c^, *MIS18A*^c^, *URB1*^c^, *ENSOCUG00000021276*^c^, *EVA1C*^c^, *CFAP298*, *SYNJ1*, *PAXBP1*^c^, *C21orf62*^c^, *ENSOCUG00000011671*, *OLIG1*, *PLCXD1*, *GTPBP6*, *ENSOCUG00000017611*
		Affx-151789209	1.14e−4	12.13	
		Affx-151983021^d^	1.34e−4	10.91	

^a^*Oryctolagus cuniculus* chromosome

^b^Bayes factor

^c^Candidate genes with relevant variants identified by whole-genome sequencing analysis

^d^SNPs that did not pass the additional tests

These 15 SNPs were further evaluated by comparing their within-population allele substitution effects and by a permutation test. The allele substitution effect estimates for the 15 SNPs did not differ significantly between lines. However, five of these SNPs, located on OCU14 and 9, did not pass the permutation test because of their high empirical p-value (EMP1). The 10 SNPs that passed the additional tests were used to perform an LD analysis and determine the ${\text{V}}_{\text{E}}$-associated genomic regions (vQTL), as described in methods (Table 1), resulting in associated LD blocks of 1.2, 1.8 and 2.4 Mb on OCU14, 10 and 3, respectively. On OCU7, no associated LD block was detected (see Additional files 2, 3, 4 and 5). Hence, vQTL were identified on OCU3 at 50.4–52.8 Mb, on OCU10 at 3.9–5.7 Mb, on OCU14 at 162–163.2, and on OCU7, close to 141,236,037 bp (Table 1).

### Candidate genes for ${\mathbf{V}}_{{\mathbf{E}}}$ of litter size

In total, 38 genes were located in the genomic regions that were associated with ${\text{V}}_{\text{E}}$ of LS (Table 1). We used WGS data of each line to identify 18,729 variants (SNVs + INDELs) in these regions (Table 2). From these, 129 were relevant (112 SNVs and 17 INDELs) based on their location in the transcription unit and/or splicing sites, which were located in 16 of the 38 genes identified in the GWAS (see Additional file 6). These 16 genes (proposed as candidate genes) are involved in biological processes related to inflammatory response, development of sensory structures, and regulation of gene expression (see Additional file 7).

**Table 2:** Classification and total number of variants (SNVs + INDELs) in each vQTL region

Region	OCU3	OCU7	OCU10	OCU14	Total
Upstream	141	21	34	328	524
5′UTR	2	0	5	0	7
Synonymous	9	2	10	34	55
Missense	1	0	6	28	35
Frameshift	0	0	0	4	4
Splicing	4	0	1	9	14
Intron	2019	267	1622	3145	7053
3′UTR	28	0	3	39	70
Downstream	193	34	57	486	770
Intergenic	3961	112	2237	3887	10,197
Total					18,729

All 129 relevant variants segregated in one of the two lines and were absent (91) or fixed (37) in the other line, except for one INDEL in the 3′UTR of the *HUNK* gene (see Additional file 6). This latter was a 1-bp deletion that was fixed in the line with a low ${\text{V}}_{\text{E}}$ of LS and absent in the line with a high ${\text{V}}_{\text{E}}$ of LS. The other variants that were fixed for the alternative allele were identified in the line with a high ${\text{V}}_{\text{E}}$ of LS in the *ITGB8*, *MIS18A*, *ENSOCUG00000021276*, and *URB1* genes, and in the line with a high ${\text{V}}_{\text{E}}$ of LS for the *HDAC9* gene (see Additional file 6). These variants could affect biological processes that are related to immune (*HDAC9*, *ITGB8,* and *HUNK*) and stress (*ENSOCUG00000021276*) responses, to regulation of gene expression (*HDAC9*, *MIS18A,* and *URB1*), and to phosphorylation of proteins (*HUNK*).

## Discussion

Our aim was to identify candidate genes and functional mutations associated with ${\text{V}}_{\text{E}}$ of litter size in rabbits. In GWAS, estimates of the effect of genomic variants on the phenotype depends on the model used [39]. In our study, we identified associated genomic regions using SMR and BMMR analyses. The SMR analysis does not consider the dependencies between SNPs, so the effects were overestimated. In addition, the number of false negatives increases when a correction such as Bonferroni is applied and variants with small effects cannot be detected. In the BMMR analysis, the shrinkage parameter of the model (π = 0.999) increases the power to detect variants with small effects but also increases the number of false positives [39]. Thus, in our study, only genomic regions that were identified by both methods were considered as candidate regions for identifying relevant genes.

Several genomic regions were associated with ${\text{V}}_{\text{E}}$ of LS (Table 1). The highlighted SNPs in these regions were further evaluated using within-population GWAS and a permutation test. However, both these tests have some limitations. The within-population GWAS, accurate estimation of the allele substitution effect was limited by the small number of individuals per population (base = 91; low = 134; high = 142), which did not represent the allele and genotype frequencies in each population. For the permutation test, the highest EMP1 of the SNPs retained in the analysis was 0.00097 (OCU14). This means that in 97 of the 100,000 permutation tests performed, the SNP was associated with ${\text{V}}_{\text{E}}$ of LS by chance under the null hypothesis. This could be due to the high level of relationship between animals in each population, which hinders elimination of true associations between genotype and phenotype.

The GWAS results were combined with WGS to identify candidate genes and functional mutations associated with ${\text{V}}_{\text{E}}$ of LS. We screened for SNVs and INDELs between the two divergent rabbit lines in the vQTL that were detected by GWAS (Table 2). A variant was considered as a potential functional mutation when it caused a missense or frameshift mutation or affected the UTR regions in the mRNAs or the splicing pattern of the transcripts. Such variants are expected to have a critical effect on the function of a gene because of a change in mRNA stability or in the amino acid sequence of the protein it encodes. Sixteen of the 38 genes identified in the GWAS contained at least one of these variants (see Additional file 6). Most of these variants segregated in one of the rabbit lines and were absent (91) or fixed (37) in the other line (see Additional file 6). The use of DNA pools for WGS allowed us to have more coverage to identify different variants between the lines with high and low ${\text{V}}_{\text{E}}$ of LS. However, the use of pools does not allow estimation of the frequency of a variant in a line, or the genotype of each animal used in the pool. For this reason, although we classified 129 variants as functional mutations, we focused on the variants that were fixed in one line and not in the other (see Additional file 6).

The 16 candidate genes identified in this study are involved in functions that are related to immune (*DOCK2*, *HDAC9*, *ITGB8,* and *HUNK*) and stress (*ENSOCUG00000021276*) responses, development of sensory structures (*FOXI1*, *FGF18*, and *EVA1C*), regulation of gene expression (*PAXBP1*, *FERD3L*, *HDAC9*, and *FOXI1*), and phosphorylation of proteins (*HUNK*), among others [see Additional file 7]. A recent study by Argente et al. [20] found differences in levels of plasma leukocytes and cortisol between the divergent rabbit lines used here but from generation 8 and showed that the line with a low ${\text{V}}_{\text{E}}$ of LS was less sensitive to infection and stress than the line with a high ${\text{V}}_{\text{E}}$ of LS. Our results confirm the importance of immune and stress responses for ${\text{V}}_{\text{E}}$ of LS through the *DOCK2*, *ITGB8, HDAC9,* and *ENSOCUG00000021276* genes. For instance, *DOCK2* is involved in the extravasation of monocytes (entry into the affected tissue) by promoting polarization of the cell membrane and remodelling of the actin cytoskeleton needed for this function. In addition, *DOCK2* controls the monocyte inflammatory response via FcyR receptors [40], such as ITGB8, through TGF-β activation [41]. The *HDAC9* gene may play a role in hematopoiesis and self-tolerance through the control of T_reg_ cells [42]. The *ENSOCUG00000021276* gene, which is orthologous to the human *MRAP (melanocortin 2 receptor accessory protein*) gene, could modulate stress response though the production of glucocorticoids in the adrenal gland but experimental analyses are needed to verify this inferred function [37].

Previous GWAS for ${\text{V}}_{\text{E}}$ in pigs and cows also identified genes that are involved in the immune response, more specifically in the inflammatory response [13–15]. Sell-Kubiak et al. [13] and Morgante et al. [14] identified genes of the HSP (heat shock protein) family to be associated with ${\text{V}}_{\text{E}}$, which regulate activation of leukocytes and protect cells against reactive-oxygen species (ROS) [43]. In mice, the candidate gene *HDAC9* regulates expression of a gene of the HSP family (*HSP70*) [42]. Wijga et al. [15] also found genes involved in the phagocytosis process to be associated with the standard deviation of milk somatic cell count in cattle, which is in line with functions related to the *DOCK2* gene [40]. Thus, there are several lines of evidence that support the importance of the immune system in the control of ${\text{V}}_{\text{E}}$.

For the other genes identified here (*FOXI1, EVAC1, FGF18,* and *HUNK*), we found no evidence in the literature that links them to a biological function associated with ${\text{V}}_{\text{E}}$. A recent study proposed to use ${\text{V}}_{\text{E}}$ as a measure of animal resilience [10], which is supported by the results of Argente et al. [20], who suggested that the line with a low ${\text{V}}_{\text{E}}$ of LS is more resilient to general stressors than the line with a high ${\text{V}}_{\text{E}}$ of LS. According to Colditz and Hine [12], animals can better maintain performance (be more resilient) when they can properly discriminate environmental stimuli from the background. In this context, the nervous system, cell receptors, and the immune system act as sensors of environmental disturbances. Thus, the immune system is required to perceive and properly respond to environmental stimuli that occur on farms, as well as correct development of the sensory organs and the neuron system [12]. Along the same line, candidate genes such as *FOXI1*, *EVAC1,* and *FGF18* would be important to develop sensory structures and parts of the nervous system. The *FOXI1* gene encodes an important transcriptional factor, which is necessary for normal development of the inner ear, with mice that lack this gene developing deafness [44]. The *EVA1C* gene is involved in the correct development of olfactory and optic sensory axons and other neural structures [45]. The growth factor FGF18 regulates development of the neural system, specifically the midbrain structure [46]. Finally, HUNK is a serine/threonine kinase, which was recently shown to be associated with control of expression of E-cadherin [47], a molecule that can act as a receptor for pathogens [48]. We identified a 1-bp deletion in the 3′UTR region of the *HUNK* gene, which was fixed in the line with a low ${\text{V}}_{\text{E}}$ of LS and absent in the line with a high ${\text{V}}_{\text{E}}$ of LS. Mutations in the 3′UTR region can affect expression of the gene and/or translation rate of the mRNA. This suggests that different levels of expression of *HUNK* between the two lines could influence ${\text{V}}_{\text{E}}$ of LS. The role of the identified candidate genes on modulation of ${\text{V}}_{\text{E}}$ of LS and, therefore, on resilient responses, requires further study to complement current evidence on the relevance of the immune system on ${\text{V}}_{\text{E}}$ [13–15, 20].

## Conclusions

A combined GWAS and WGS analysis allowed us to identify 16 new candidate genes that carry 129 putative functional mutations that are associated with ${\text{V}}_{\text{E}}$ of LS in rabbits. These findings provide support for the control of ${\text{V}}_{\text{E}}$ of LS through regulation of the immune system and suggest that development of the nervous system and sensory structures may also be important to modulate animal resilience. This study advances our understanding of the genetic background of ${\text{V}}_{\text{E}}$. However, further studies are needed to validate the true effect of the putative functional mutations in these genes on ${\text{V}}_{\text{E}}$ of LS, as well as the relationship of ${\text{V}}_{\text{E}}$ with animal resilience.

## Supplementary information

**Additional file 1.** Principal component analysis applied to the genotype data. Representation of the first (PC1) and second (PC2) components for the genotypes in the base population (red) and in the high (blue) and low (green) selection lines for environmental variance (${\text{V}}_{\text{E}}$) of litter size (LS).

**Additional file 2.** Linkage disequilibrium of SNPs on OCU3 at 49–53 Mb. Representation of the linkage disequilibrium (LD) in the associated genomic region on OCU3. SNPs in this region were plotted according to their Bayes factor ($BF$). The colours of the SNPs indicate their LD with the SNP with the highest $BF$ in this region (highlighted with a black triangle). Colours between red and green indicate an r^2^ between 1 and 0.5. Colours between green and blue indicate an r^2^ between 0.5 and 0. Genes in this region are plotted at the bottom of the graph according to their position on the genome.

**Additional file 3.** Linkage disequilibrium of SNPs on OCU7 at 140–142 Mb. Representation of the linkage disequilibrium (LD) in the associated genomic region on OCU7. SNPs in this region were plotted according to their Bayes factor ($BF$). The colours of the SNPs indicate their LD with the SNP with the highest $BF$ in this region (highlighted with a black triangle). Colours between red and green indicate an r^2^ between 1 and 0.5. Colours between green and blue indicate an r^2^ between 0.5 and 0. Genes in this region are plotted at the bottom of the graph according to their position on the genome.

**Additional file 4.** Linkage disequilibrium of SNPs on OCU10 at 3–7 Mb. Representation of the linkage disequilibrium (LD) in the associated genomic region on OCU10. SNPs in this region were plotted according to their Bayes factor ($BF$). The colours of the SNPs indicate their LD with the SNP with the highest $BF$ in this region (highlighted with a black triangle). Colours between red and green indicate an r^2^ between 1 and 0.5. Colours between green and blue indicate an r^2^ between 0.5 and 0. Genes in this region is plotted at the bottom of the graph according to their position on the genome.

**Additional file 5.** Linkage disequilibrium of SNPs on OCU14 at 161–164 Mb. Representation of the linkage disequilibrium (LD) in the associated genomic region in OCU14. SNPs in the region were plotted according to their Bayes factor ($BF$). The colours of the SNPs indicate their LD with the SNP with the higher $BF$ in this region (highlighted with a black triangle). Colours between red and green indicate r^2^ between 1 and 0.5. Colours between green and blue indicate r^2^ between 0.5 and 0. Genes in this region was plotted at the bottom of the graphic according their position in the genome.

**Additional file 6.** Candidate genes and their biological function based on the GeneCards and Ensembl databases.

**Additional file 7.** Total number of relevant variants identified in the associated genomic regions for the environmental variance of litter size. INDELs and SNVs identified by WGS analysis in a UTR or splicing region or with a missense effect. For each variant, the position is indicated according to the chromosome (OCU) and base pair (bp) location. REF show the allele in the reference genome (*Oryctolagus cuniculus* v2.0.96) and ALT the alternative variant identified in the rabbit lines. Low and High show the allelic distribution in each line where 0 indicates the reference allele and 1 the alternative allele.

## Data Availability

Data are available upon request to the corresponding author.
