# Supplementary figures and images for: Identification of functional mutations associated with environmental variance of litter size in rabbits
Source: Genet Sel Evol. 2020 May 6;52:22. doi: 10.1186/s12711-020-00542-w (PMC7203823; doi:10.1186/s12711-020-00542-w)

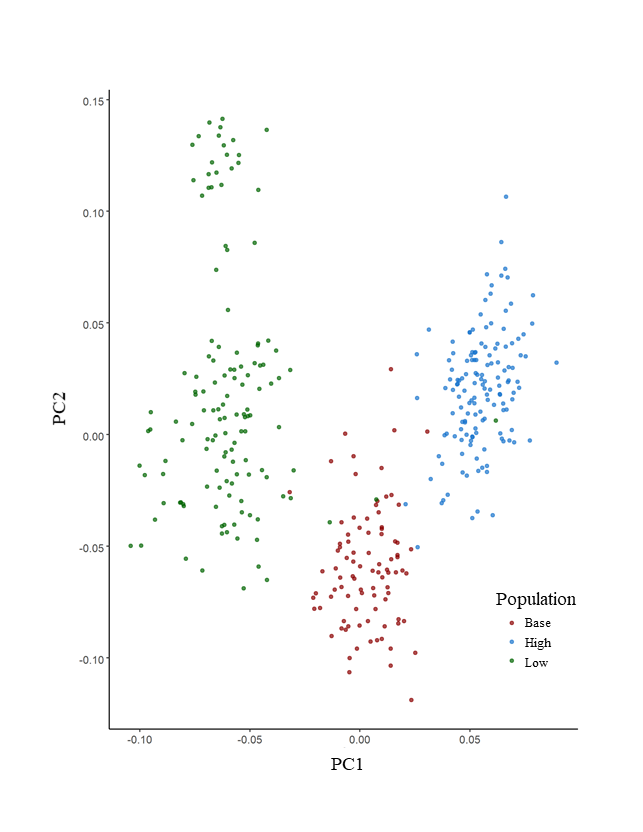

Supplement: Supplementary file 1 — Additional file 1. Principal component analysis applied to the genotype data. Representation of the first (PC1) and second (PC2) components for the genotypes in the base population (red) and in the high (blue) and low (green) selection lines for environmental variance (${\text{V}}_{\text{E}}$VE) of litter size (LS). [file 12711_2020_542_MOESM1_ESM.tif]

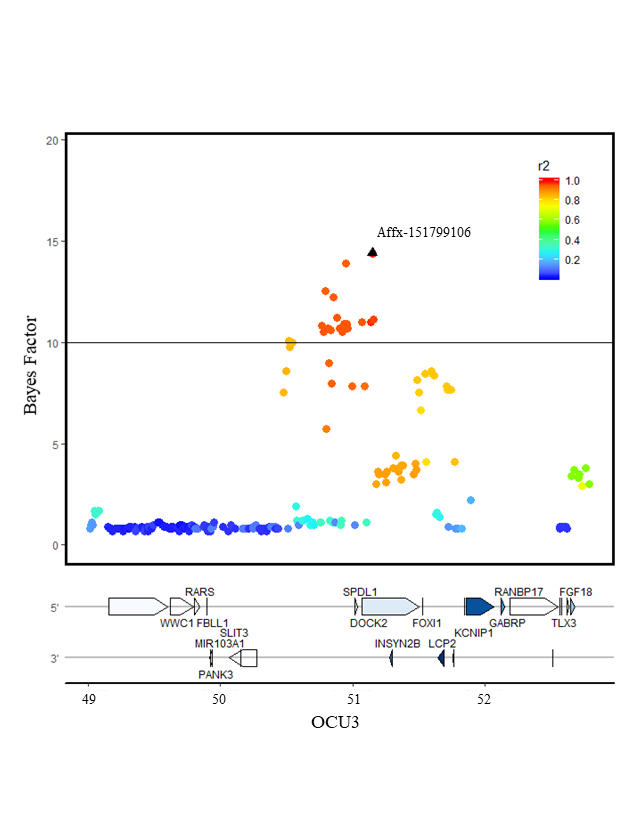

Supplement: Supplementary file 2 — Additional file 2. Linkage disequilibrium of SNPs on OCU3 at 49–53 Mb. Representation of the linkage disequilibrium (LD) in the associated genomic region on OCU3. SNPs in this region were plotted according to their Bayes factor ($BF$BF). The colours of the SNPs indicate their LD with the SNP with the highest $BF$BF in this region (highlighted with a black triangle). Colours between red and green indicate an r2 between 1 and 0.5. Colours between green and blue indicate an r2 between 0.5 and 0. Genes in this region are plotted at the bottom of the graph according to their position on the genome. [file 12711_2020_542_MOESM2_ESM.tif]

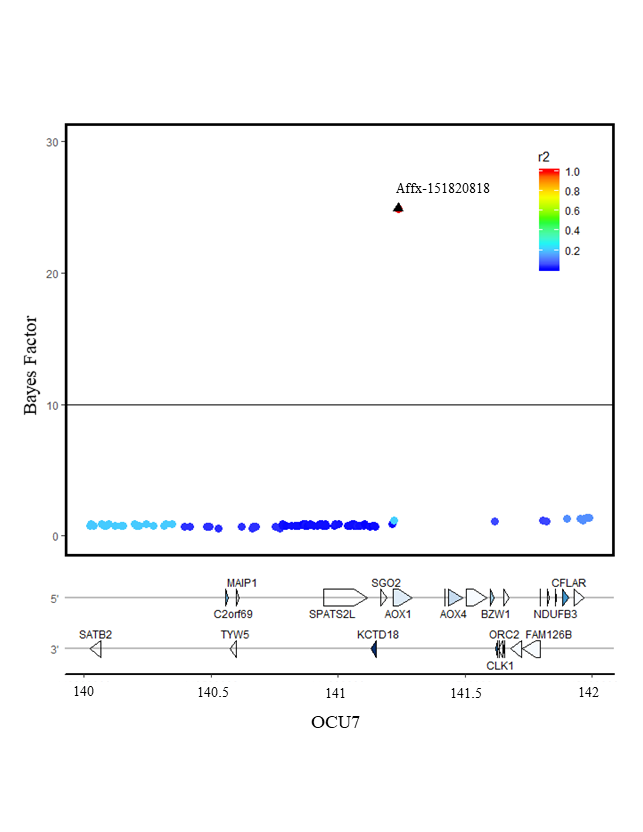

Supplement: Supplementary file 3 — Additional file 3. Linkage disequilibrium of SNPs on OCU7 at 140–142 Mb. Representation of the linkage disequilibrium (LD) in the associated genomic region on OCU7. SNPs in this region were plotted according to their Bayes factor ($BF$BF). The colours of the SNPs indicate their LD with the SNP with the highest $BF$BF in this region (highlighted with a black triangle). Colours between red and green indicate an r2 between 1 and 0.5. Colours between green and blue indicate an r2 between 0.5 and 0. Genes in this region are plotted at the bottom of the graph according to their position on the genome. [file 12711_2020_542_MOESM3_ESM.tif]

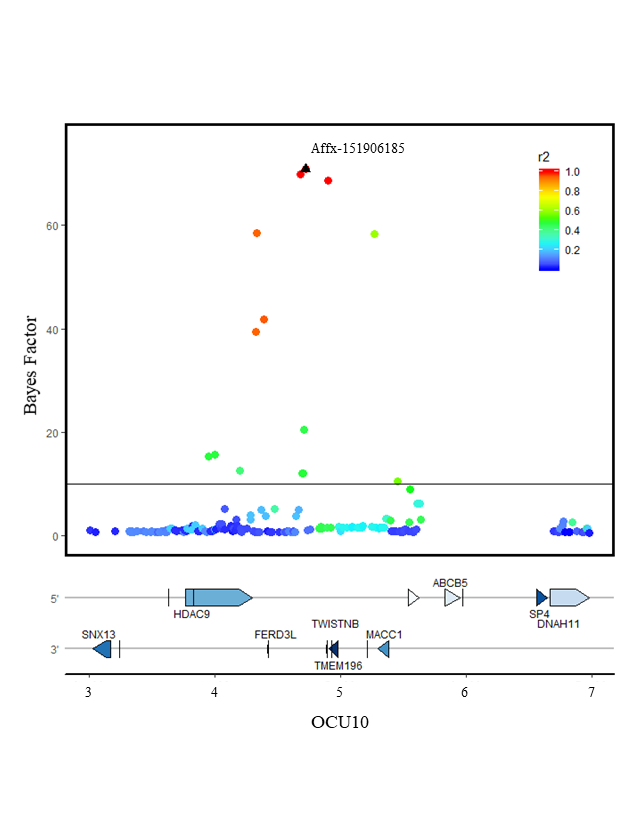

Supplement: Supplementary file 4 — Additional file 4. Linkage disequilibrium of SNPs on OCU10 at 3–7 Mb. Representation of the linkage disequilibrium (LD) in the associated genomic region on OCU10. SNPs in this region were plotted according to their Bayes factor ($BF$BF). The colours of the SNPs indicate their LD with the SNP with the highest $BF$BF in this region (highlighted with a black triangle). Colours between red and green indicate an r2 between 1 and 0.5. Colours between green and blue indicate an r2 between 0.5 and 0. Genes in this region is plotted at the bottom of the graph according to their position on the genome. [file 12711_2020_542_MOESM4_ESM.tif]

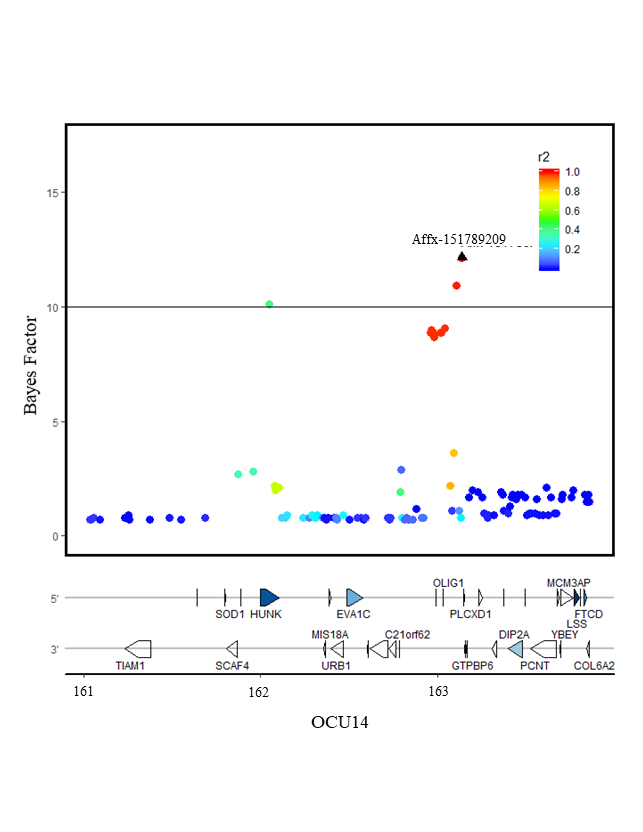

Supplement: Supplementary file 5 — Additional file 5. Linkage disequilibrium of SNPs on OCU14 at 161–164 Mb. Representation of the linkage disequilibrium (LD) in the associated genomic region in OCU14. SNPs in the region were plotted according to their Bayes factor ($BF$BF). The colours of the SNPs indicate their LD with the SNP with the higher $BF$BF in this region (highlighted with a black triangle). Colours between red and green indicate r2 between 1 and 0.5. Colours between green and blue indicate r2 between 0.5 and 0. Genes in this region was plotted at the bottom of the graphic according their position in the genome. [file 12711_2020_542_MOESM5_ESM.tif]
